# MicroRNA-101a enhances trabecular bone accrual in male mice

**DOI:** 10.1038/s41598-022-17579-0

**Published:** 2022-08-03

**Authors:** Amel Dudakovic, Sofia Jerez, Padmini J. Deosthale, Janet M. Denbeigh, Christopher R. Paradise, Martina Gluscevic, Pengfei Zan, Dana L. Begun, Emily T. Camilleri, Oksana Pichurin, Farzaneh Khani, Roman Thaler, Jane B. Lian, Gary S. Stein, Jennifer J. Westendorf, Lilian I. Plotkin, Andre J. van Wijnen

**Affiliations:** 1 Department of Orthopedic Surgery, Mayo Clinic, Rochester, MN USA; 2 Department of Biochemistry & Molecular Biology, Mayo Clinic, Rochester, MN USA; 3 Department of Anatomy, Cell Biology & Physiology, Indiana University School of Medicine, Indianapolis, IN USA; 4 Mayo Clinic Graduate School of Biomedical Sciences, Mayo Clinic, Rochester, MN USA; 5 Center for Regenerative Medicine, Mayo Clinic, Rochester, MN USA; 6 Department of Orthopedic Surgery, School of Medicine, Second Affiliated Hospital of Zhejiang University, Hangzhou, China; 7 Department of Orthopedic Surgery, School of Medicine, Shanghai Tenth People’s Hospital Affiliated to Tongji University, Shanghai, China; 8 Department of Biochemistry, University of Vermont, Burlington, VT USA; 9 Indiana Center for Musculoskeletal Health, Indiana University School of Medicine, Indianapolis, IN USA; 10 Richard L Roudebush VA Medical Center, Indianapolis, IN USA

**Keywords:** Cell biology, Molecular biology

## Abstract

High-throughput microRNA sequencing was performed during differentiation of MC3T3-E1 osteoblasts to develop working hypotheses for specific microRNAs that control osteogenesis. The expression data show that miR-101a, which targets the mRNAs for the epigenetic enzyme Ezh2 and many other proteins, is highly upregulated during osteoblast differentiation and robustly expressed in mouse calvaria. Transient elevation of miR-101a suppresses Ezh2 levels, reduces tri-methylation of lysine 27 in histone 3 (H3K27me3; a heterochromatic mark catalyzed by Ezh2), and accelerates mineralization of MC3T3-E1 osteoblasts. We also examined skeletal phenotypes of an inducible miR-101a transgene under direct control of doxycycline administration. Experimental controls and mir-101a over-expressing mice were exposed to doxycycline in utero and postnatally (up to 8 weeks of age) to maximize penetrance of skeletal phenotypes. Male mice that over-express miR-101a have increased total body weight and longer femora. MicroCT analysis indicate that these mice have increased trabecular bone volume fraction, trabecular number and trabecular thickness with reduced trabecular spacing as compared to controls. Histomorphometric analysis demonstrates a significant reduction in osteoid volume to bone volume and osteoid surface to bone surface. Remarkably, while female mice also exhibit a significant increase in bone length, no significant changes were noted by microCT (trabecular bone parameters) and histomorphometry (osteoid parameters). Hence, miR-101a upregulation during osteoblast maturation and the concomitant reduction in Ezh2 mediated H3K27me3 levels may contribute to the enhanced trabecular bone parameters in male mice. However, the sex-specific effect of miR-101a indicates that more intricate epigenetic mechanisms mediate physiological control of bone formation and homeostasis.

## Introduction

Osteogenesis is mediated by the dynamic activation and suppression of gene expression programs in osteoblasts that produce a collagenous mineralized matrix^1–9^. The production and deposition of bone-specific extracellular matrix proteins is controlled by epigenetic mechanisms that modulate the accessibility of transcription factors to gene loci in chromatin^1,10^. Genetic studies have established that many distinct epigenetic regulators contribute to skeletal development and bone formation in vivo, including enzymes and proteins that control DNA methylation, as well as histone acetylation and methylation^10–12^. Beyond chromatin-related mechanisms, bone-specific gene expression is also controlled by post-transcriptional epigenetic events mediated by microRNAs (miRNAs) that regulate stability and/or translation of osteoblast-related mRNAs^5,13^.

The importance of miRNAs in control of bone formation and homeostasis in vivo is evidenced by mouse models incapable of producing mature miRNAs due to conditional knock-out of the endonuclease Dicer^14^. It has been challenging to investigate the functional roles for specific miRNA loci because of redundancy between miRNA isoforms that are encoded by multiple related miRNA gene loci or nearly identical gene copies^13^. The latter has been addressed in part by strategies that target multiple miRNAs at once using compound gene knockouts or decoys^15,16^. Many studies have focused on the identification of miRNAs that regulate expression of important signaling pathways and transcription factors in cell culture models for osteoblastogenesis^17–26^. Among the latter studies, several related themes have emerged. First, principal gene regulators that control osteoblast differentiation are targeted by more than one miRNA^21,27^. For example, the bone master regulator Runx2 is controlled by more than ten distinct miRNAs that collectively may control lineage commitment in mesenchymal progenitor cells^19,21,27^. Other studies have shown that miRNA gene clusters, which encode distinct sets of miRNAs with different targets, may collectively regulate signaling pathways required for osteogenesis^22,23^. These studies reflect the complexity of miRNA mediated regulatory networks in which each miRNA has a large number of potential mRNA targets and that each mRNA target can interact with many distinct miRNAs. This molecular diversity permits different levels of regulatory interdependence between signaling pathways and transcription factors and supports negative and positive feedback loops that can either stimulate or attenuate cellular processes like osteoblastogenesis^5,13,28^.

In this study, we examined miRNAs that are upregulated during calvarial osteoblast differentiation. Among these miRNAs we detected miR-101a, a microRNA that has multiple targets including the epigenetic regulator Ezh2^29–32^. The latter epigenetic enzyme suppresses osteoblast differentiation^11,33–36^ as well as supports lineage progression and self-renewal of mesenchymal stromal cells^37–40^. To assess whether this nexus between mir-101a and Ezh2 impacts bone formation, we performed in vivo gain of function studies with miR-101a using an inducible transgenic approach. Here we show that miR-101a has sex-dependent post-natal effects on trabecular bone parameters.

## Results

### Changes in miRNA expression during differentiation of MC3T3 pre-osteoblasts

To assess changes in the non-coding transcriptome of miRNAs during osteoblastogenesis, microRNA-sequencing (miRNA-seq) was performed using total RNA isolated during differentiation of MC3T3 E1 subclone 4 (referred to here as MC3T3) pre-osteoblasts. Because the MC3T3 cell line was originally derived from mouse calvaria, total RNA samples from frontal and parietal bones derived from neo-natal (i.e., 2–3 day old pups) female mice were evaluated in parallel for in vivo relevance. Based on our previous RNA-seq assessment^35^ (data not shown), MC3T3 E1 subclone 4 cells express genes located on the Y chromosome (e.g., Kdm5d and Uty), suggesting that these cells are derived from male mice. Of the 1,418 miRNAs detected by our sequencing platform, 168 miRNAs exhibit very robust expression with counts per million (CPM) values exceeding one hundred (CPM > 100) when averaged across five MC3T3 differentiation time points (days 0, 6, 10, 17, and 24) (Suppl Table 1). While some miRNAs are differentially expressed, expression of other miRNAs remains relatively constant during the MC3T3 differentiation time course. Unlike miRNA-seq analysis using calvaria that were sequenced with bones from three animals (biological triplicates), each individual time point obtained during the MC3T3 time course was analyzed as a single miRNA-seq run of a pooled biological triplicate sample because inter-sample variation for established cell lines is remarkably minor in our experience. Although no statistical analysis was possible on individual two-way sample comparisons for MC3T3 cells during the time course, the consistency of these data is reflected by matrix analysis (hierarchical clustering in heatmap) and curve fitting (time course graph) (see below).

**Figure 1:**
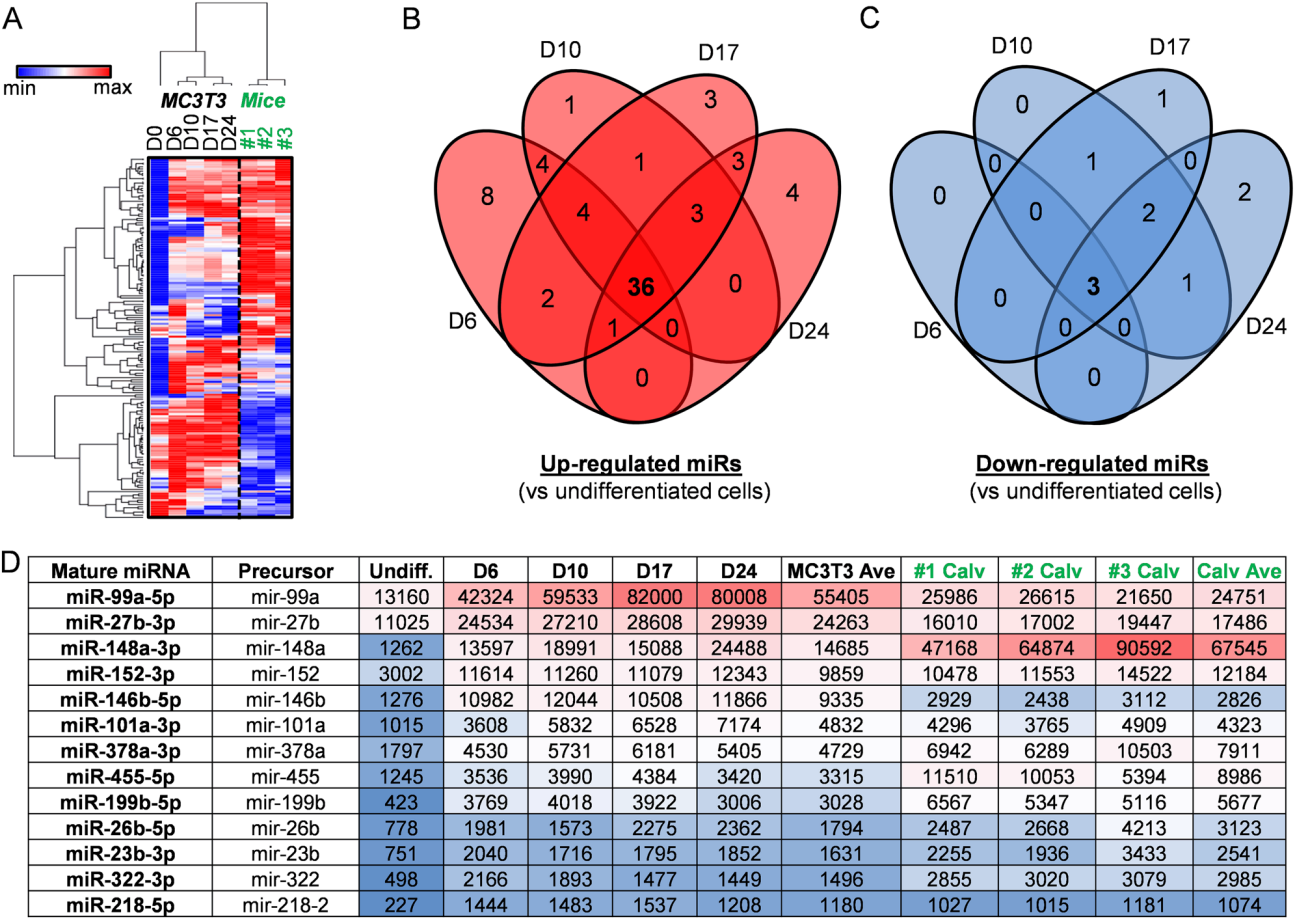
miRNA expression patterns in differentiating MC3T3 cells and primary mouse calvaria. miRNA-Seq analysis was performed on RNA derived from MC3T3 cells undergoing osteogenic differentiation (days 0, 6, 10, 17, and 24) and primary female mouse calvaria (animal identification #1, #2, and #3). Hierarchical clustering of miRNAs robustly expressed (CPM > 100 across five samples) in differentiating MC3T3 cells (n = 168) (**A**). The distribution of miRNAs in primary mouse calvaria (n = 3) are also included for in vivo relevance. Venn diagram analysis of robustly expressed miRNAs (n = 168) that are up-regulated (**B**) and down-regulated (**C**) across various differentiation time points (days 6, 10, 17, and 24) relative to undifferentiated cells (day 0) in MC3T3 cells (fold change (FC) > 2). Table summarizing commonly up-regulated miRNAs (see Panel B) that are highly expressed in MC3T3 cells and primary mouse calvaria [CPM > 1000 on average in MC3T3 cells (n = 5) and primary mouse calvaria (n = 3)] (**D**).

Hierarchical clustering analysis of robustly expressed miRNAs reveals separation between differentiating MC3T3 cells and mouse calvaria (Fig. 1A). Interestingly, these analyses also reveal three temporal sub-clusters within MC3T3: undifferentiated (day 0), early differentiation states (days 6 and 10), and late differentiation states (days 17 and 24). To identify robustly expressed miRNAs that may regulate osteogenic differentiation, additional analysis focused on the identification of miRNAs that are differentially expressed during osteoblast differentiation of MC3T3 cells. Of the robustly expressed miRNAs (n = 168), 36 miRNAs are up-regulated (Fig. 1B) and three are down-regulated (Fig. 1C) across all four differentiation time points when compared to undifferentiated MC3T3 cells (fold change (FC) > 2) (Suppl Table 2). To narrow the search of miRNAs that may regulate osteogenesis, miRNAs induced on average above 1,000 CPM in both MC3T3 cells (in vitro model) and primary mouse calvaria (in vivo relevance) (Suppl Table 2 and Fig. 1D) were selected for further analysis. Of these 13 miRNAs, we considered miR-101a-3p of greatest interest, because it was shown to suppress Ezh2^29,41^, which is an epigenetic enzyme that mediates trimethylation of lysine 27 on histone 3 (H3K27me3). Our previous work established that repression of Ezh2 promotes osteogenic differentiation in vitro, stimulates bone formation in vivo, and protects against bone loss due to estrogen depletion^33,35,37,42^. Therefore, the present studies address the role of miR-101a on osteogenesis in vitro and bone formation in vivo.

### miR-101 modulates Ezh2 activity and enhances osteogenic differentiation of MC3T3 cells

**Figure 2:**
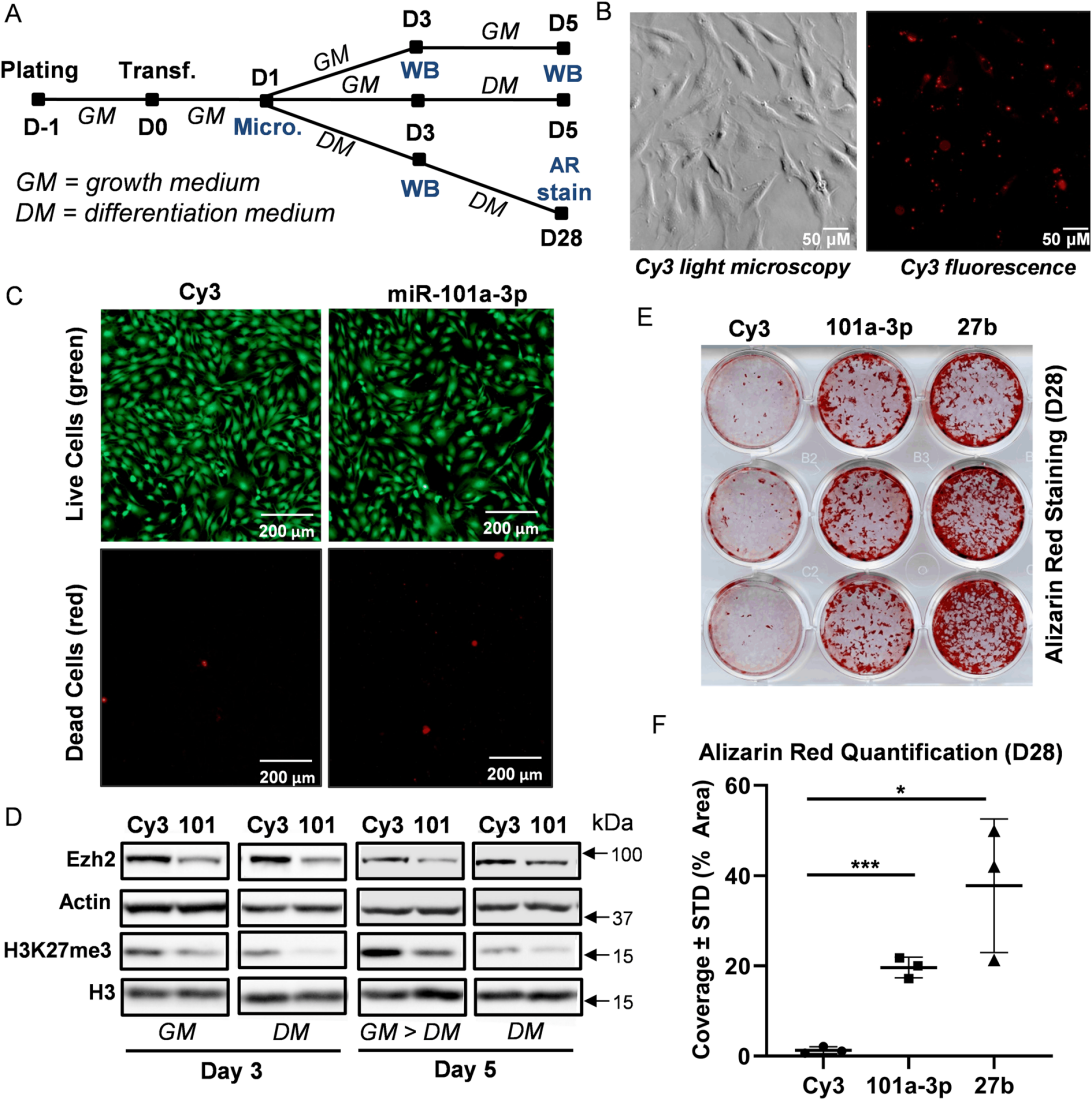
miR-101 targets Ezh2 and stimulates mineral deposition in differentiating MC3T3 cells. Experimental set-up of MC3T3 transfection and differentiation (**A**). Cells were plated and transfected in growth medium. Cells were then maintained in growth medium (GM) and differentiation medium (DM) for indicated times. Media were changed every two to three days (*Micro.* Microscopy, *WB* Western blotting, *AR stain* alizarin red staining). Representative light and fluorescence microscopy images of Cy3 dye-labeled pre-miRNA negative control transfected MC3T3 cells one day after transfection (**B**). Representative live (green) and dead (red) fluorescence microscopy images of Cy3 negative control and miR-101a-3p transfected MC3T3 cells (**C**). Western blot analysis of MC3T3 cell lysates transfected with Cy3 negative control and miR-101-3p (**D**). Alizarin red staining (**E**) and quantification (**F**) of differentiating MC3T3 cells (day 28) transfected with CY3 control, miR-101a-3p, and miR-27b [n = 3, mean ± standard deviation (STD)]. *p < 0.05 and ***p < 0.001. All experiments were repeated at least three times.

We first sought to determine whether miR-101 over-expression can modulate Ezh2, H3K27 methylation, and osteogenic differentiation in mouse derived MC3T3 pre-osteoblasts (Fig. 2A). One day after transfection, Cy3 (negative control pre-miR) was detected by fluorescence microscopy (Fig. 2B). Live and dead cell staining revealed similar viability in Cy3 and miR-101a-3p transfected MC3T3 cells (Fig. 2C). Western blotting analysis indicates a reduction in Ezh2 protein and H3K27me3 levels three and five days after transfection of miR-101a-3p in MC3T3 cells grown in either basal or differentiation medium (Fig. 2D). The levels of the cytoplasmic protein β-actin (gene symbol ACTB) and total levels of the nuclear protein histone 3 (H3) are indistinguishable between Cy3 and miR-101a-3p transfected cells indicating that the gel lanes were loaded equally. Our results validates previous findings for the miR-101a-3p/Ezh2 nexus initially observed in non-bone related cancer cell types^29,41^, osteosarcoma^43^ and during osteogenic differentiation of mesenchymal stromal cells^44^, and extends these findings to osteoblastic MC3T3 cells.

To assess the impact of miR-101a on osteogenesis, MC3T3 cells were differentiated upon transfection with Cy3, miR-101a-3p or miR-27b, which was also up-regulated during osteogenic differentiation (Fig. 1) and previously shown to stimulate osteoblast differentiation^45,46^. Over-expression of either miR-101a-3p or miR-27b enhances osteogenic differentiation of MC3T3 pre-osteoblasts as shown by alizarin red staining (Fig. 2E,F). Taken together, our evidence shows that miR-101 suppresses Ezh2 and H3K27me3 levels and enhances osteogenic commitment of MC3T3 pre-osteoblasts. The finding that miR-101 regulation of Ezh2 and osteogenesis in vitro is sufficiently robust in different biological contexts provides a solid experimental foundation for in vivo studies on the role miR-101 in bone formation.

### Establishing miR-101 over-expression in bone tissue

**Figure 3:**
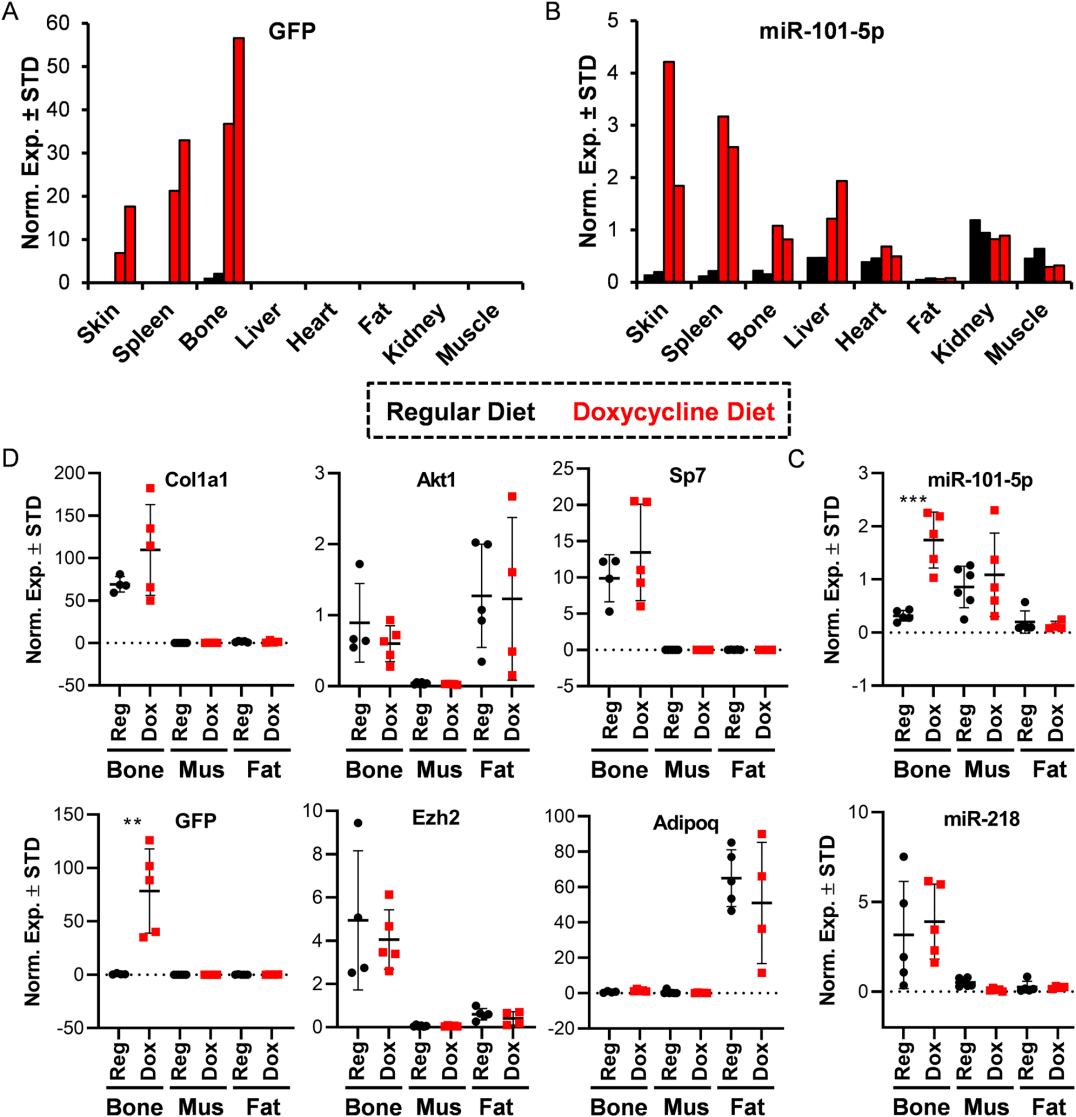
Establishment of miR-101a over-expressing mouse model in vivo. Three-week-old R26/101a male and female mice were maintained on regular (reg) or fed a doxycycline-supplemented (dox) diet for five weeks. Indicated tissues were assessed by RT-qPCR analysis. Administration of doxycycline is anticipated to induce miR-101a and GFP expression. An expression screen for GFP (**A**) and miR-101-5p (**B**) in eight tissues derived from eight-week-old female mice (n = 2). Quantitative expression of Col1a1, GFP, Akt1, Ezh2, Sp7/Osx, and Adipoq (**C**) and miR-101-5p and miR-218 (**D**) in bone (femora), skeletal muscle (thigh area), and fat (visceral) of eight-week-old male mice (n = 4 to 5, mean ± STD). **p < 0.01 and ***p < 0.001. We note that miR-218 was arbitrarily included as a representative miRNA to indicate selectivity in the differences in expression of miR-101-5p across tissues. MiRNA expression levels are presented using miR-103a-3p as internal control for normalization.

We evaluated the role of miR-101a on bone formation by establishing a transgenic mouse colony through mouse embryonic stem cells from the National Cancer Institute^47^. In this transgenic mouse model, expression of miR-101a is achieved using a gene cassette that was inserted in a transcriptionally permissive region downstream of the 3’UTR of the Col1a1 gene (Suppl Fig. 1). This cassette contains a tetracycline (tet)-responsive element (TRE-tight; Tet Operator/TetO) upstream from a green fluorescent protein (GFP) with a 3′ untranslated region containing the sequences for miR101a (i.e., embedded within the pre-miRNA scaffold for human miR-30). This primary transgene is activated by a secondary transgene expressing a doxycycline-inducible tet-transactivator (tTA) from the Rosa26 locus (Rosa26/tTA). Three-week-old mice heterozygous for both the mir-101a over-expression cassette and the Rosa26/tTA locus were maintained on a regular doxycycline-rich diet for five weeks to assess miR-101a and GFP expression (Fig. 3). In a RT-qPCR screen of eight tissues, GFP expression is evident in skin, spleen, and bone (femora) in eight-week-old female mice on doxycycline diet (Fig. 3A). Because GFP is not normally expressed in mice, the GFP/miR-101a transgene model should represent a zero-background system in the absence of doxycycline. Nevertheless, we detected low levels of GFP expression due to ‘leaky’ transcription from the TRE-tight promoter in tissues from mice maintained on a regular diet see (black bars in bone). Similar to GFP, mir-101a expression is enhanced by doxycycline administration in skin, spleen, and bone (Fig. 3B). Unlike GFP, miR-101a is also up-regulated in liver; because the transgene transcript encodes both RNAs, this finding may perhaps reflect the relative stabilities of small miRNAs versus longer mRNAs.

**Figure 4:**
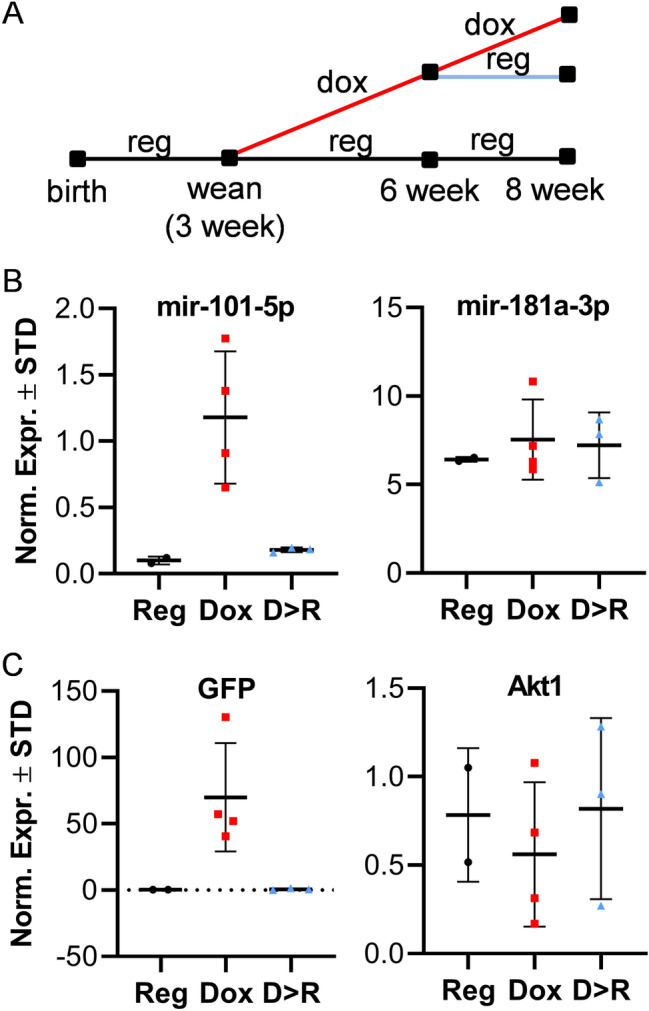
In vivo induction of miR-101a is reversible. Three-week-old R26/101a male mice were fed regular (reg, R) or doxycycline-supplemented diet (dox, D) to assess the reversibility of miR-101a induction in vivo. Schematic representation of the mouse feeding regiment beginning at three and ending at eight weeks of age (**A**). Expression of miR-101-5p and miR-181a-3p (**B**) as well as GFP and Akt1 (**C**) in femora of eight-week-old mice (n = 2 to 4); miR-181a was arbitrarily included as a representative miRNA with a different expression pattern to reflect selectivity in the upregulation of miR-101-5p. MiRNA expression levels are presented using miR-103a-3p as internal control for normalization.

To confirm utility of this mouse model for examination of skeletal phenotype and for bone-related studies, expression of both miR-101a and GFP was also assessed in bones (femora) and two additional mesenchymal tissues (muscle and fat) from a larger cohort of male mice. GFP expression was significantly enhanced in the femora of male mice upon doxycycline-administration (Fig. 3D). As anticipated, mRNAs for Col1a1 and Sp7 (encoding the bone-specific transcription factor Osterix) are both expressed in bone, while Adipoq mRNA (encoding adiponectin) is enriched in fat. Interestingly, while Ezh2 mRNA is unchanged by the doxycycline diet, basal Ezh2 mRNA levels are higher in bone tissues when compared to muscle and fat. Similar to Ezh2, expression of the housekeeping gene Akt1 is not affected by doxycycline although its levels vary between mesenchymal tissues. Importantly, miR-101a levels are significantly up-regulated (5.5-fold) in femora of mice on doxycycline diet (Fig. 3C), but the levels of an independent miRNA, miR-218, are not affected. To determine if miR-101a over-expression is reversible in bone tissue*,* male mice were fed regular and/or doxycycline-supplemented diet (Fig. 4A). Like the initial cohorts (Fig. 3), a five-week doxycycline diet enhances expression of both miR-101a (Fig. 4B) and GFP (Fig. 4C) in eight-week-old male femora. Interestingly, replacement of doxycycline with regular diet (D > R) at six weeks abolishes expression of miR-101a and GFP levels but does not affect transcript levels of miR-181a and Akt1 (negative controls). In summary, while not tissue-specific, the GFP/miR-101a mouse model allows for inducible and reversible up-regulation of miR-101a in bone tissues with GFP expression as a useful reporter.

### miR-101a over-expression stimulates bone formation in male mice

To assess the impact of miR-101a over-expression on bone formation, two control groups and the experimental group of mice were placed on doxycycline diet from inception until sacrifice^47–49^. The two control groups are: R26 control (heterozygous for tTA/Rosa26 locus) and miR-101a control (heterozygous for mir-101a over-expression cassette). The experimental group is referred to as R26/101a or miR-101a over-expressing mice (heterozygous for both the GFP/mir-101a over-expression cassette and tTA/Rosa26 locus). Together these groups adequately account for the effects of miR-101a overexpression and doxycycline exposure, which can modulate bone formation^50–52^.

**Figure 5:**
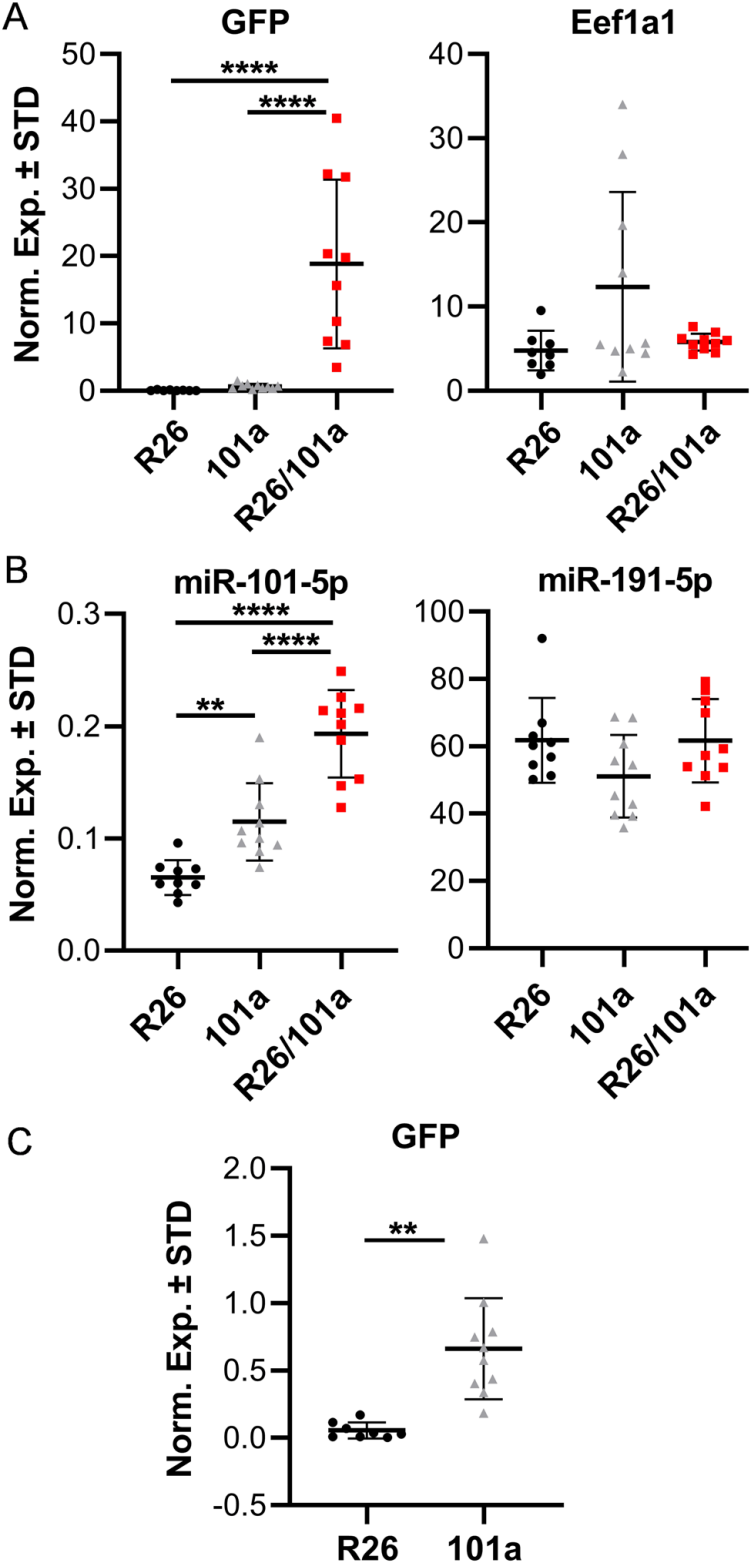
miR-101a expression is induced in male mice. Femora (left) were collected from R26 control, miR-101a control, and miR-101a over-expressing (R26/101) male mice treated with doxycycline from inception to sacrifice (8 weeks). RNA (**A**) and miRNA (**B**) RT-qPCR analysis of all three mouse cohorts as well as RT-qPCR assessment for GFP in two control groups (**C**) (same values as in A) (n = 8 to 10, mean ± STD) are shown. **p < 0.01 and ****p < 0.0001. We note that miR-191-5p was arbitrarily included as a representative miRNA to reflect selectivity in modulations in the expression of miR-101-5p. MiRNA expression levels are presented using miR-103a-3p as internal control for normalization.

To assess GFP and mir-101a levels, RT-qPCR analysis was performed on femoral RNA derived from the three male cohort groups. When compared to both control groups, GFP mRNA levels are significantly enhanced in the miR-101a over-expressing mice (Fig. 5A). The levels of Eef1a1, a stably expressed housekeeping gene, remain constant among the three mouse groups. Similar to GFP mRNA levels, miR-101-5p is up-regulated in bone tissues of miR-101a over-expressing mice as expected (Fig. 5B). For comparison, the levels of miR-191-5p are constant across the three mouse groups. Interestingly, expression analysis reveals a difference in the levels of miR-101-5p (Fig. 5B) and GFP mRNA (Fig. 5C) between the two control groups; as noted above, detection of any mRNA may be less sensitive than any miRNA because the latter tend to be more stable. Thus, while miR-101a is robustly induced in the miR-101a over-expressing mice, it should also be noted that miR-101a is also elevated in the 101a control group when compared to the R26 control group, suggesting leaky transcription from the miR-101a over-expression cassette.

**Figure 6:**
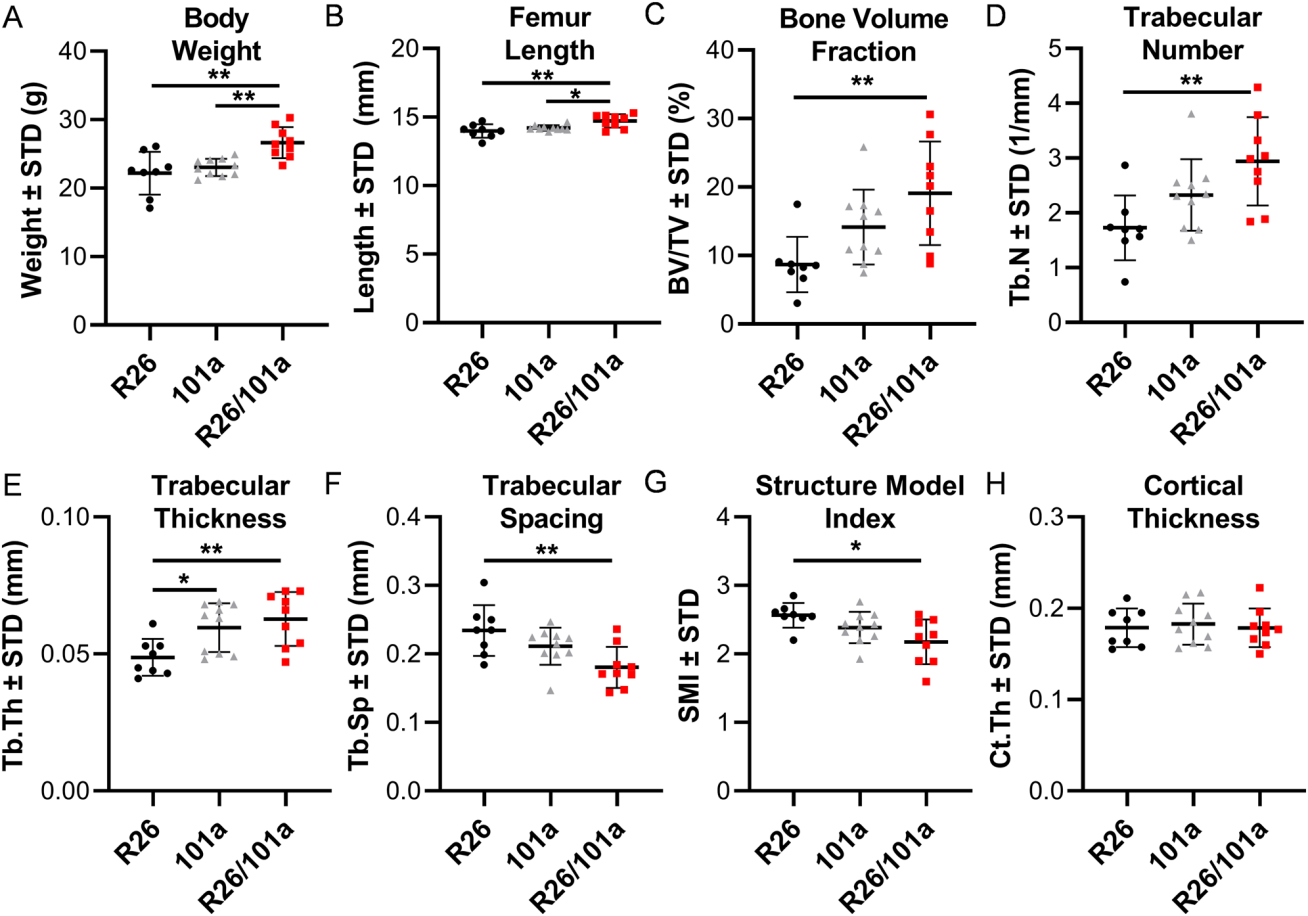
miR-101a over-expression enhances trabecular bone mass in male mice. R26 control, miR-101 control, and miR-101a over-expressing (R26/101) male mice were treated with doxycycline from inception to sacrifice (8 weeks). The figure depicts body weight (**A**), femur length (**B**), trabecular bone parameters (**C–G**), and cortical thickness (**H**) as assessed by µCT (n = 8 to 10, mean ± STD). *p < 0.05 and **p < 0.01.

**Figure 7:**
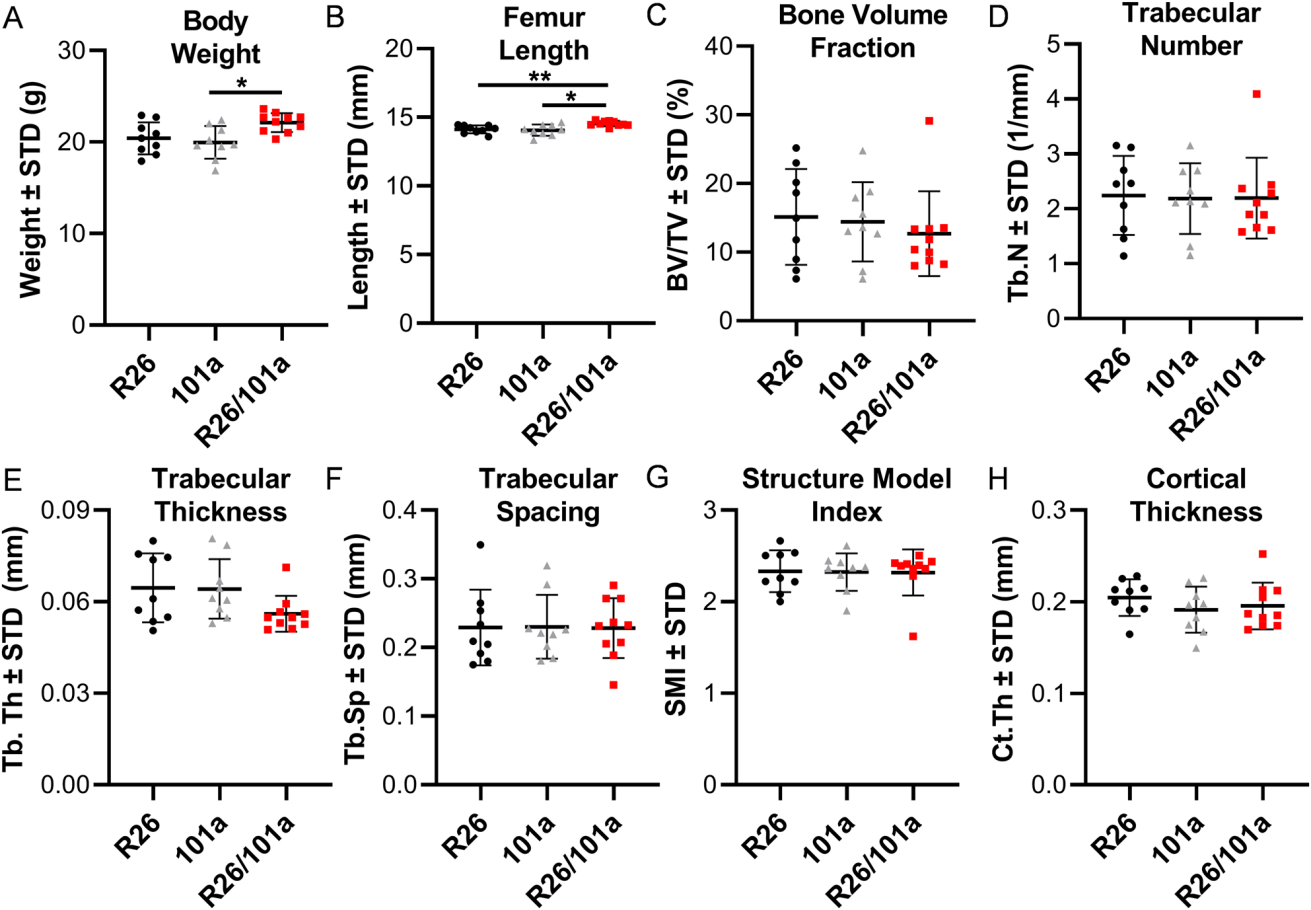
miR-101a over-expression does not alter bone mass in female mice. R26 control, miR-101 control, and miR-101a over-expressing (R26/101) female mice were treated with doxycycline from inception to sacrifice (8 weeks). The figure depicts body weight (**A**), femur length (**B**), trabecular bone parameters (**C–G**), and cortical thickness (**H**) as assessed by µCT (n = 9 to 10, mean ± STD). *p < 0.05 and **p < 0.01.

Overexpression of miR101a in male mice increases femoral length with a commensurate increase in body weight when compared to both R26 and miR-101a control mice (Fig. 6A,B). Female mice exhibit a similar overall phenotype with miR-101a dependent changes in weight and femoral length phenotype, except that the body weight of female miR-101a over-expressing mice was only statistically different from miR-101a controls, but not from R26 controls (Fig. 7A,B). Skeletal analyses using μCT reveals increases in trabecular bone volume fraction, trabecular number, and trabecular thickness, while a decrease in trabecular spacing and structure model index is observed in male miR-101a over-expressing mice when compared to R26 control mice (Fig. 6C–G). The miR-101a control mice are similar to R26 control mice (except for increased trabecular thickness), but the μCT parameters are not significantly different between miR-101a controls and miR-101a over-expressing mice. Of interest, when assessing body weight, femur length, and trabecular bone parameters, the phenotype of miR-101a control mice is consistently between R26 controls and miR-101a over-expressing mice. The latter is consistent with ‘leaky’ transcription of the transgenic cassette as noted above (see Figs. 3 and 5). Thus, the elevated expression of the miR-101a transgene in the miR-101a controls may explain the lack of statistical difference between miR-101a controls and miR-101a over-expressing male mice (see Fig. 5). In contrast to male mice, no differences in trabecular bone parameters measured by µCT are observed among the three groups in female mice (Fig. 7C–G). Further, overexpression of miR-101a did not alter cortical bone geometry in the femoral mid-diaphysis for either male (Fig. 6H) or female (Fig. 7H) mice. Thus, μCT results collectively indicate that inducible transgenic expression of miR-101a during post-natal skeletal development alters bone parameters.

**Figure 8:**
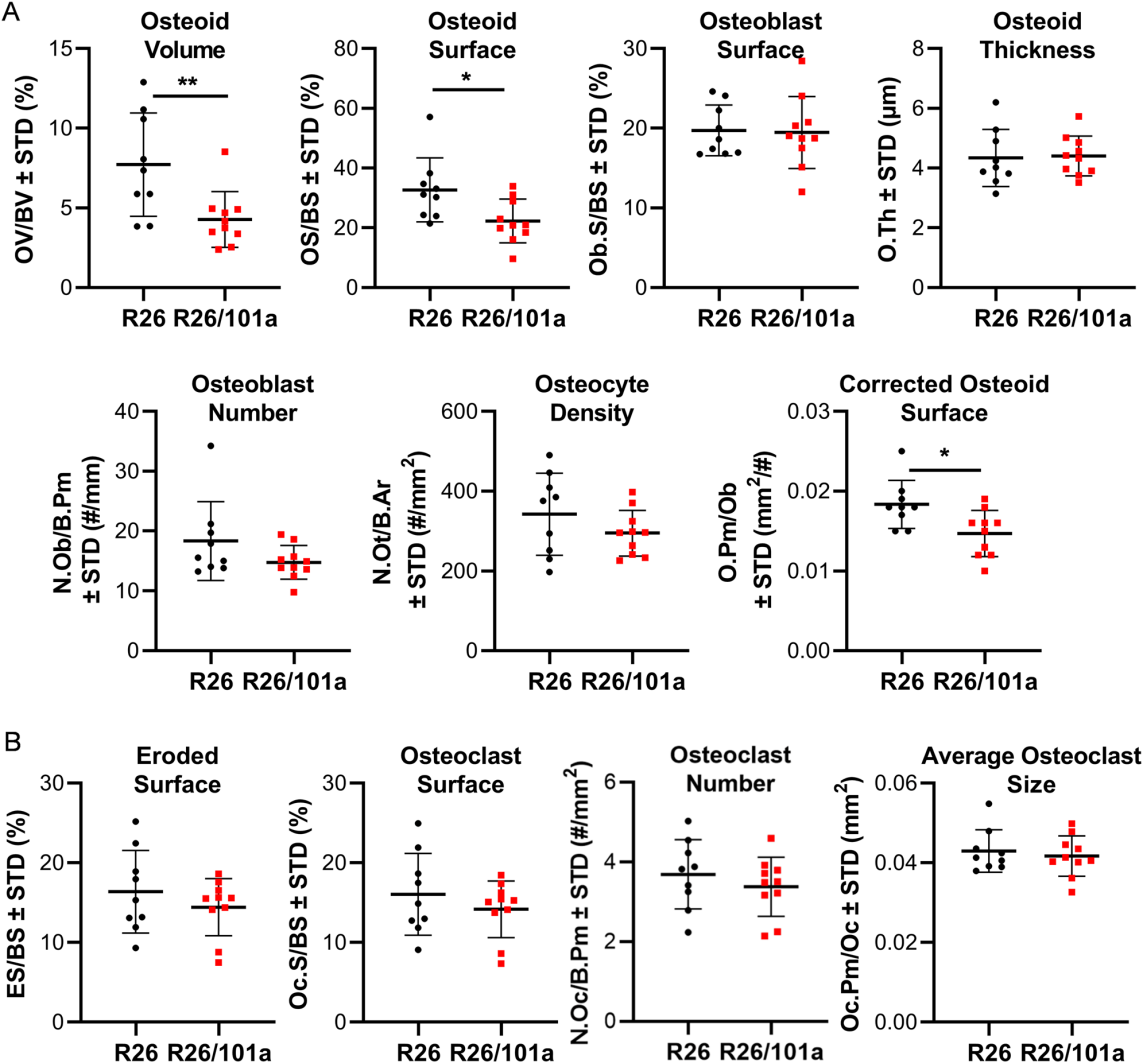
Histomorphometric alterations upon miR-101a over-expression in male mice. Femora (right) were collected from R26 control and miR-101a over-expressing (R26/101a) male mice treated with doxycycline from inception to sacrifice (8 weeks). The figure depicts histomorphometry measurements of von Kossa/McNeal-stained (**A**) and TRAPase/Toluidine blue-stained (**B**) sections derived from of sagittal femora slices (n = 9 to 10, mean ± STD). *p < 0.05 and **p < 0.01.

**Figure 9:**
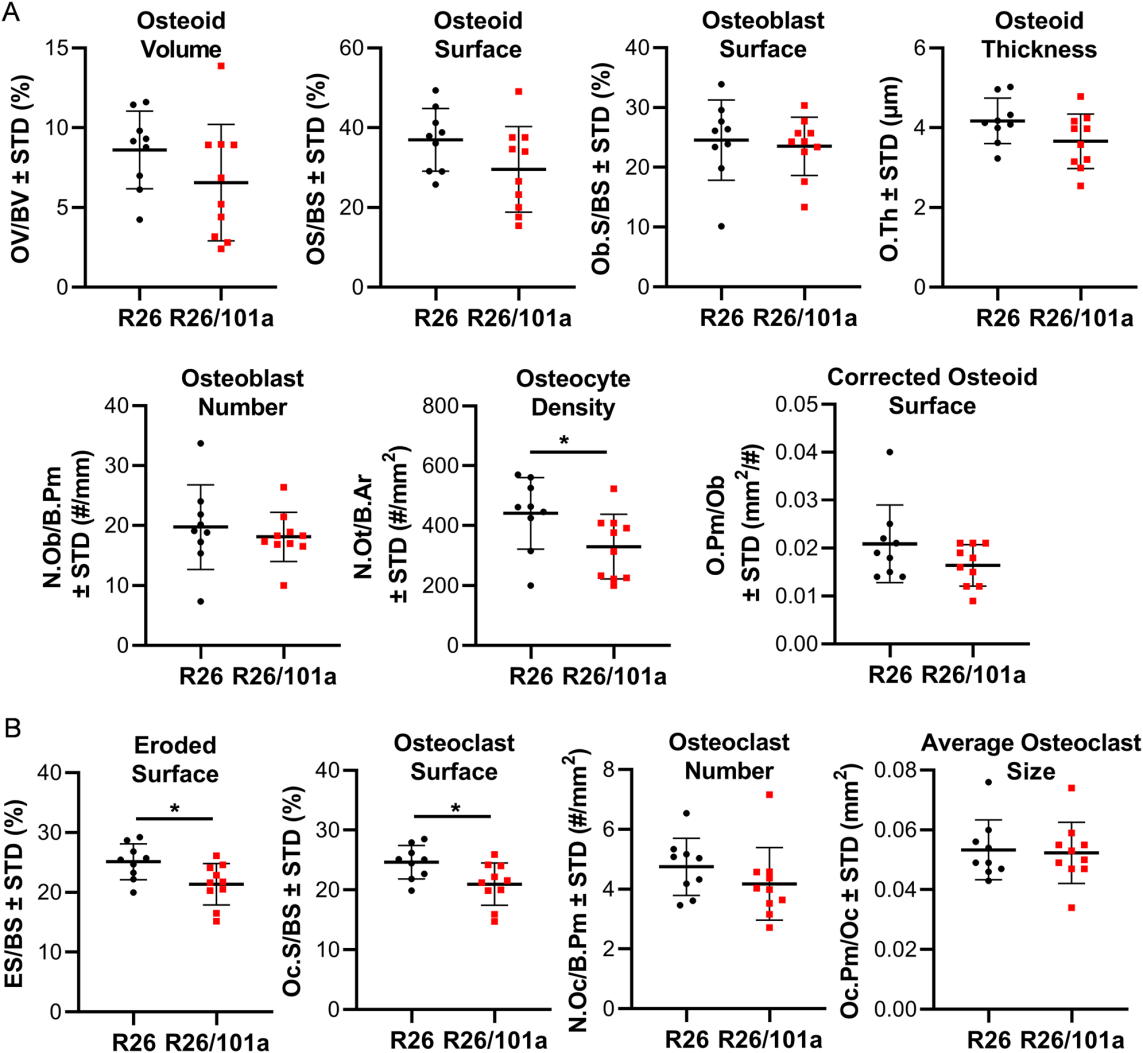
Histomorphometric alterations upon miR-101a over-expression in female mice. Femora (right) were collected from R26 control and miR-101a over-expressing (R26/101a) female mice treated with doxycycline from inception to sacrifice (8 weeks). The figure depicts histomorphometry measurements of von Kossa/McNeal-stained (**A**) and TRAPase/Toluidine blue-stained (**B**) sections derived from of sagittal femora slices (n = 9 to 10, mean ± STD). *p < 0.05.

**Figure 10:**
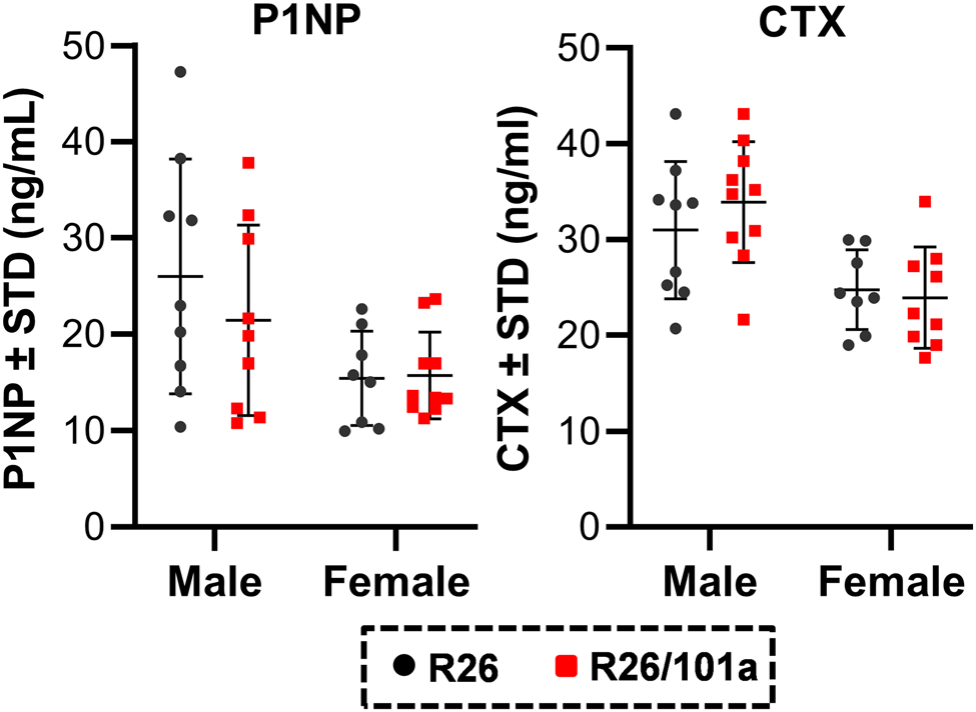
Circulating bone marker levels are not altered by miR-101a over-expression male and female mice. Terminal serum samples were collected from male and female R26 control and miR-101a over-expressing (R26/101a) mice treated with doxycycline from inception to sacrifice (8 weeks). Circulating levels of P1NP and CTX were assessed by commercial ELISA kits.

Because miR-101a control is leaky and permits a limited level of miR-101a expression even in the absence of tetracycline-dependent transcriptional activator (tTA), histomorphometric analyses were performed on femora derived from R26 control and miR-101a overexpressing mice (Suppl Fig. 2). Static histomorphometric analyses performed on cancellous bone of the distal femur showed that osteoid volume and surface covered by osteoid are significantly decreased in bones from male mice, whereas the number and surface of osteoblasts do not vary, resulting in a significant decrease in the corrected osteoid surface per osteoblast in miR-101a over-expressing male mice when compared to R26 control male mice (Fig. 8). On the other hand, osteoclast number and surface, as well as eroded surface and osteocyte density are similar between R26 control and miR-101a over-expressing male mice. However, miR-101a over-expression does not alter osteoid or osteoblastic parameters but results in a small but significant decrease in osteocyte density, and in osteoclast and eroded surface in cancellous bone of distal femurs when compared to R26 control in female mice (Fig. 9). These results do not exclude the possibility that increased bone mass due to decreased bone resorption could still become evident at a later age, but this prediction has not yet been tested. Regardless of the local changes in bone geometry and cell number/surface that reflect the dynamics of bone homeostasis, the overall remodeling state is not altered by miR-101a overexpression, as evidenced by lack of changes in circulating levels of the bone formation marker P1NP (i.e., procollagen type I N-terminal propeptide) and the resorption marker CTX (i.e., cross-linked C-telopeptide of type I collagen) in either male or female mice (Fig. 10). We cannot rule out the possibility that an imbalance favoring bone formation occurred at an earlier age (or during development), and that the animals reached a new steady state in which formation and resorption are similar to those of control mice at the time of euthanasia. Nevertheless, these data demonstrate that persistent miR-101a induction (from inception to 8 weeks) stimulates trabecular bone formation in male mice.

### Trabecular bone formation is refractory to miR-101a induction post-weaning in male mice

**Figure 11:**
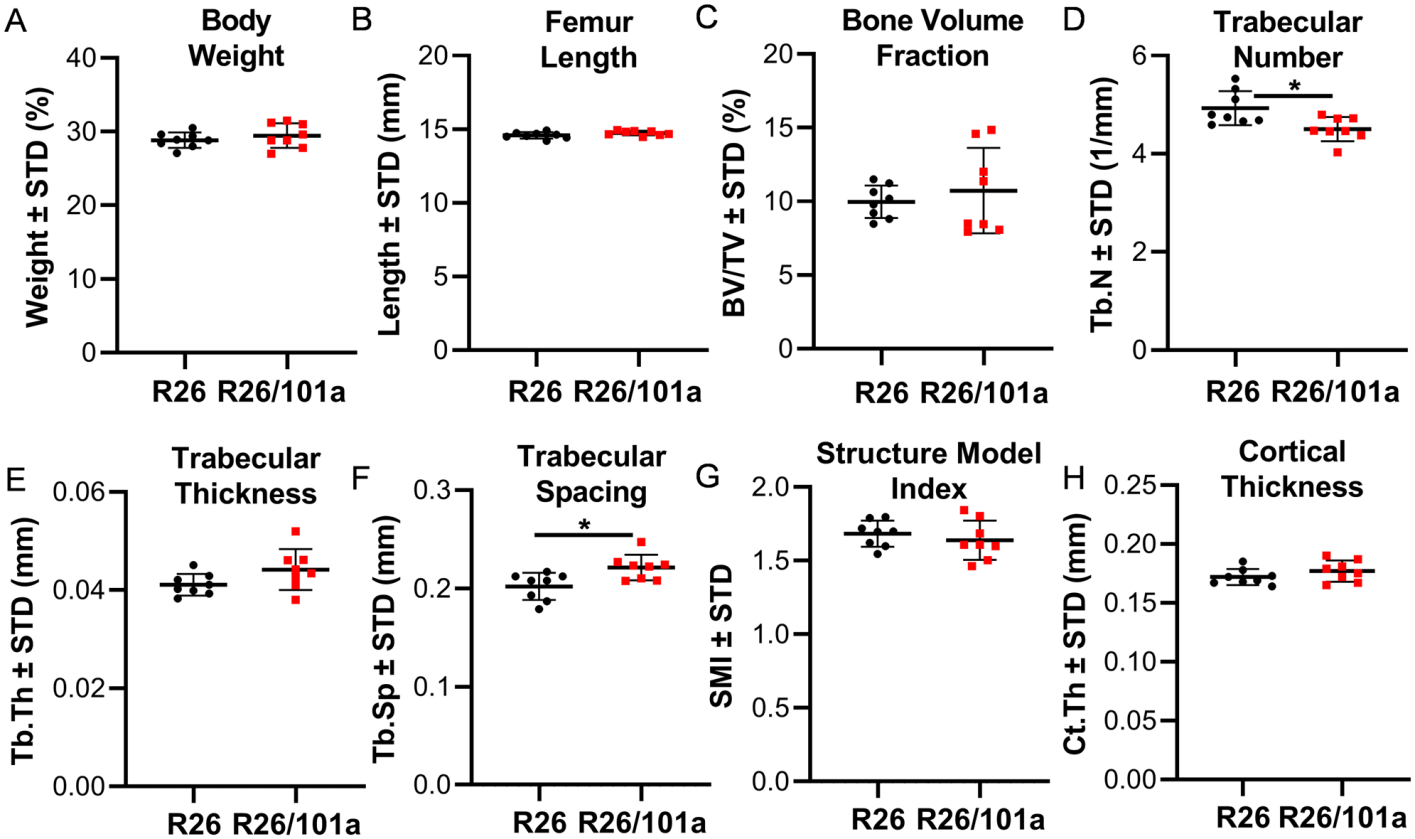
Post-weaning induction of miR-101a is insufficient to increase bone mass in male mice. R26 control and miR-101a over-expressing (R26/101) male mice were treated with doxycycline from 3 to 12 weeks of age. The figure depicts whole body weight (**A**) as well as femur length (**B**), trabecular bone parameters (**C–G**), and cortical thickness (**H**) as assessed by µCT (n = 8, mean ± STD). *p < 0.05.

To assess the impact of miR-101a on bone formation after weaning, three-week-old male mice from R26 control and miR-101 over-expressing cohorts were placed on a doxycycline rich diet until early skeletal maturity (twelve weeks). No differences were observed in body weight (Fig. 11A) and femora length (Fig. 11B) between control and miR-101a over-expressing mice. A small but significant reduction in trabecular number (Fig. 11D) and an increase in trabecular spacing (Fig. 11F) are apparent, but the trabecular bone volume fraction (Fig. 11C), trabecular thickness (Fig. 11E), trabecular structural model index (Fig. 11G), and cortical thickness (Fig. 11H) are similar between R26 control and miR-101a over-expressing mice. Taken together, inducible over-expression of miR-101a after weaning is not sufficient to enhance bone formation in male mice.

## Discussion

Alterations in the expression of epigenetic molecules, including miRNAs and epigenetic enzymes, during mesenchymal progenitor commitment and osteoblast lineage progression can reveal epigenetic events that maintain progenitors in an undifferentiated state or may stimulate their commitment into more differentiated states. Our current approach utilized microRNA-sequencing to identify miRNAs that are differentially expressed in MC3T3 cells and may regulate osteogenic differentiation and bone formation. Results from our microRNA-Seq expression screen revealed numerous differentially expressed miRNAs during osteogenic differentiation of MC3T3 cells. Our strategy prioritized miRNAs that are expressed in calvaria and robustly up-regulated during osteoblast differentiation. Of the thirteen miRNAs that met both of these criteria, miR-101a is a non-coding RNA based suppressor that is known to target Ezh2 mRNA and other mRNAs. Of these regulatory interactions, the miR-101a/Ezh2 nexus has been established in many different cancer cells^29,41,53^, and may operate ubiquitously in many other cell types.

While previous studies have firmly established that Ezh2 is a key target for miR-101a in osteosarcoma^43^ and during osteogenic differentiation of mesenchymal stromal cells^32^, functional miRNA/mRNA interactions operative in one cell type may not occur in other cell types. For example, our previous studies have revealed cell type-specific differences in the potency of miRNAs (e.g., miR-23a and miR30c) to control distinct osteogenic or chondrogenic transcription factors (i.e., Runx2 and Trps1)^21,27^. In view of cell type specific miRNA effects, it is reassuring that our data formally demonstrate that miR-101a indeed controls Ezh2 levels in osteoblastic MC3T3 cells.

There are many predicted targets for miR-101a that may influence bone cell physiology. We focused our interpretations of the physiological function of miR-101a on Ezh2, because Ezh2 is a broad epigenetic suppressor of osteogenesis that may account for many of the biological effects mediated by miR-101a. The observation that miR-101a upregulation correlates with decreased Ezh2 expression during osteoblast differentiation is consistent with an expanding number of studies from our group and others on the role of Ezh2 as a mediator of mesenchymal stem cell maintenance and an epigenetic suppressor of osteogenic differentiation^35–38,42,54–63^. These studies collectively revealed that Ezh2 suppresses osteogenic differentiation and inhibition of this epigenetic enzyme is bone-anabolic and osteo-protective. Mechanistically, loss of Ezh2 function may enhance osteogenic pathways, including PTH, WNT, and BMP signaling. The present work supports and refines this model by invoking the idea that these osteogenic pathways are controlled by the Ezh2/miR-101a nexus because (i) miR-101a is reciprocally regulated relative to Ezh2 and (ii) miR-101a gain-of-function reduces Ezh2 levels in MC3T3 osteoblasts. Similar to knock-down and knock-out strategies or small molecule inhibition for Ezh2 that have bone anabolic potential^35,37,63,64^, miR-101a may thus provide a natural small non-coding RNA-based mechanism by which Ezh2 (and perhaps other suppressors of osteogenesis directly targeted by miR-101a) can be targeted in osteoblast progenitor cells to stimulate osteogenic differentiation and bone formation.

Support for the impact of miR-101a on Ezh2 and osteogenic differentiation is provided by results from studies in which miR-101a-3p was transiently over-expressed in MC3T3 cells. Similar to the initial studies on the effects of miR-101a on human EZH2 in cancer cells^29,41^, over-expression of miR-101a-3p suppresses Ezh2 protein levels in mouse MC3T3 osteoblasts cells maintained in either basal or osteogenic medium. Importantly, our studies also reveal a robust suppression of the H3K27me3 heterochromatin mark that is catalyzed by Ezh2. This finding indicates that Ezh2 is indeed a major cellular target of miR-101a and that there is mechanistic interplay between translational suppression of the multiple putative mRNA targets of miR-101a and reversal of Ezh2 mediated transcriptional suppression. Biologically, miR-101a-3p over-expression enhances alizarin red staining of MC3T3 cell cultures. Our findings are further supported by previous studies by Wang and colleagues who demonstrated that miR-101a stimulates osteogenic commitment of human bone-marrow derived mesenchymal stromal cells^59^. Similar to miR-101a-3p, over-expression of miR-27b, another highly up-regulated miRNA in differentiating MC3T3s, stimulates MC3T3 differentiation as demonstrated by enhanced alizarin red staining. The involvement of miR-27b on osteogenic differentiation has been previously established in human fetal osteoblasts and may involve modulation of WNT signaling. Together, the pro-osteogenic functions of miR-101a-3p and miR-27b may converge on WNT signaling by the targeting of Ezh2 to alleviate suppressive epigenetic marks^35,42,59^ and targeting of WNT modulators (SFRP1)^45,46^, respectively.

To assess the impact of miR-101a in bone formation in vivo, we generated mice from transgenic embryonic stem cells in which miR-101a and GFP are expressed under control of a tetracycline/doxycycline responsive element (TRE)^47^ that is genomically incorporated into transcriptionally permissive locus immediately downstream from the Col1a1 gene. By design, the over-expression of miR-101a is inducible by the presence of tTA (expressed from the Rosa26 locus) and doxycycline in many tissues; expression of the miR-101a transgene is primarily limited by the physiological availability of the doxycycline in cells and tissues. Characterization of this transgenic doxycycline inducible TRE/GFP/miR-101a mouse model demonstrates that doxycycline administration induces miR-101a levels in bone tissues. The up-regulation is reversible as doxycycline removal restores miR-101a levels to endogenous levels. Our findings thus validate the use of this type of transgenic mouse model as a valuable tool for assessing the overall physiological function of miRNAs in bone and other tissues.

To our knowledge, this study provides the first evidence showing that enhancing miR-101a expression stimulates bone formation in vivo. However, one of the limitations of our study is the broad tissue distribution of miR-101a expression. We noted that beyond enhanced levels in bone, miR-101a levels are also elevated as fully expected in other tissues (e.g., skin, spleen, and liver). These findings thus confirm that induction of miRNAs from the doxycycline inducible transgene occurs in a range of tissues consistent with the original design of the mouse model. As has been noted previously, this transgenic miRNA expression model can in principle be further refined by expressing the doxycycline-response tTA protein from a stringently regulated tissue specific promoter^47^. However, even if tissue specific expression was achieved, in vivo observations could still be confounded by the findings that miRNA levels can circulate in bodily fluids and may alter expression of targeted proteins at distal tissues^5,65–67^. Another limitation of our study is that our analysis of skeletal phenotypes is restricted (e.g., static versus dynamic bone morphometry, as well as no analysis of bone resorption, bone repair, steroid hormone depletion, or metabolic disruption). Considering the current pleiotropic effects of the current miR-101a transgene model, these studies will be more informative with a more refined mouse model that specifically expresses miR-101a only in committed osteoblasts or osteocytes.

To assess the impact of miR-101a over-expression on bone formation, control and experimental mice were placed on doxycycline diet from inception until sacrifice (blood stream in utero, mother’s milk, and consumption of doxycycline diet)^47–49^. We observe increases in overall body weight and bone length in miR-101a over-expressing male and female mice. However, enhanced trabecular bone formation as measured by µCT analysis is only observed in male, but not female mice. Other sex-specific differences included a reduction in osteoid-related parameters, which may stem from accelerated bone mineralization, in males while females exhibited a reduction in osteoclast related parameters. Interestingly, miR-101a over-expression after weaning (3 to 12 weeks) did not alter the weight and bone length. Minor changes in trabecular bone parameters are observed and may suggest an inhibitory role for miR-101a post-weaning as indicated by a reduction in trabecular number and increase trabecular spacing. Altogether, our in vivo studies reveal that enhanced miR-101a expression stimulates bone formation when up-regulated in utero, but these effects are no longer observed when miR-101a induction is induced after weaning. These findings suggest that there is a principal role for miR-101a in early stages of development and that miR-101a may accelerate bone growth during endochondral ossification to increase bone length in males and females, as well as trabecular bone formation in male mice. The physiological reasons for the sex-related and developmental-stage dependent differences in bone phenotypes remain speculative at present. The complexity of developmental, molecular, physiological, and genetic considerations precludes the design of definitive experiments that would address uncertainties in the interpretation at this time.

While miR-101 has many predicted mRNA targets beyond Ezh2, the relationship between Ezh2 and miR-101 appears to be quite critical for cell growth and differentiation. The miR-101 dependent inhibition of Ezh2 reflects the dynamic and intricate interdependence between two distinct epigenetic regulatory events involving miR-101a suppressed translation (non-genomic) and Ezh2 suppressed transcription (epigenomic). Earlier studies by Varambally and colleagues revealed that expression of Ezh2 is enhanced upon genomic loss of miR-101^29^. Consistent with our studies showing that Ezh2 is suppressed during osteogenic differentiation^35,37,42^, our present study and the work by Wang and colleagues^32^ demonstrate that Ezh2 is a miR-101 target in differentiating MSCs and committed pre-osteoblasts. Thus, miR-101 may enhance osteogenic differentiation by suppressing the expression and activity of Ezh2, perhaps in parallel with physiological bone stimulatory effects of miR-101a on other mRNA targets and in distinct bone-related cell types (e.g., MSCs, osteoblasts, osteocytes, and osteoclasts). In support of our findings showing enhanced bone formation upon miR-101 induction, miR-101 over-expression in human MSCs enhances new bone formation in a mouse calvarial defect model^32^. Further corroborated by the work of Huang and colleagues^59^, a model has emerged in which miR-101 and Ezh2 form a reciprocal negative feedback loop to regulate and fine-tune their expression levels and activities.

In conclusion, our present work and previous studies collectively demonstrate that miR-101 promotes osteogenic differentiation and bone formation in vivo. While the functional role of miR-101 in osteogenesis is intricately intertwined with Ezh2 levels, future studies may provide insights into Ezh2-independent functions of miR-101 and whether circulating levels of miR-101 may contribute to the observed bone-anabolic properties of this microRNA.

## Experimental procedures

### MC3T3 cell culture and osteogenic differentiation

MC3T3 E1 sc4 murine calvarial osteoblasts^68^ were purchased from American Type Culture Collection and maintained in α-minimal essential medium without ascorbic acid (Gibco) containing 10% fetal bovine serum (Atlanta Biologicals), 100 units/ml penicillin (Gibco), and 100 μg/ml streptomycin (Gibco) in cell culture incubators (37 °C and 5% CO_2_). For experiments, cells were plated at 10,000 cells/cm^2^ in maintenance medium. Osteogenic differentiation was induced the next day by the addition of 50 μg/ml ascorbic acid (Sigma) and 4 mM β-glycerol phosphate (Sigma). Alternatively, cells were transfected one day after plating and maintained in growth medium or differentiated in osteogenic medium as described in text.

### Isolation of tissues for RNA assessment

Parietal and frontal bones *(*calvaria) were harvested from three 2–3 days old C57BL/6 Prrx1-Cre + female pups (i.e., #1, #2, and #3 are pup identification numbers)^69^. For these studies, RNA from Prxx1-Cre + mice was used to reduce the number of animals as this RNA was previously generated for another study^37^. To our knowledge, no studies to date have reporter a phenotype in Prrx1-Cre + , which alleviates concerns related to potential miRNA changes in Prxx1-Cre + relative to C57BL/6 wild type mice. The calvaria were then washed in cold phosphate buffered saline (PBS), incubated in collagenase digestion medium (αMEM, 2 mg/ml collagenase type II, and 0.005% trypsin) for 20 min at 37 °C, washed in PBS, snap-frozen in liquid nitrogen, and stored at − 80 °C until further processing^37,57^. Tissues from eight-week-old mice were freshly harvested, snap-frozen in liquid nitrogen, and stored at − 80 °C until further processing.

### Transfection of miRNA mimics

MC3T3 E1 sc4 cells were transfected with miRNA mimics as described^35,37,70^. Briefly, cells were seeded in 6- or 12-well plates in maintenance medium (10,000 cells/cm^2^). The next day, control (Cy3 dye-labeled Pre-miRNA Negative Control, AM17120, Thermo Fisher) and miRNA mimics, miR-101a-3p (MC11414, Thermo Fisher) and miR-27b (MC10750, Thermo Fisher), were transfected using RNAiMAX as instructed by the manufacturer (Invitrogen). Cells were subsequently cultured as described in the text.

### RNA isolation and RT-qPCR analysis

Total RNA was isolated using the Direct-zol™ RNA kit (Zymo Research). Before isolation, calvaria (2-to-3-day old mice) and other tissues (eight-week-old mice) were homogenized for 30 s in ice-cold QIAzol using the Ultra Turrax T25 tissue homogenizer (IKA). RNA was quantified using the NanoDrop 2000 spectrophotometer (Thermo Fischer Scientific).

For mRNA analysis, RNA was reverse transcribed into cDNA by the SuperScript III First-Strand Synthesis System (Invitrogen). Gene levels were assessed using real-time PCR using the QuantiTect SYBR Green PCR Kit (Qiagen) and the CFX384 Real-Time System machine (BioRad). Transcript levels were quantified using the 2^ΔΔCt^ method and normalized to the housekeeping gene Gapdh (set at 100). Primer pairs are shown in Suppl Table 3.

For miRNA quantification, miRNAs were reverse transcribed into cDNA using the Mir-X miRNA First-Strand Synthesis Kit as instructed (Takara). Similar to mRNA, miRNA levels were determined using QuantiTect SYBR Green PCR Kit and the CFX384 Real-Time System machine. miRNA quantification was assessed using a universal reverse primer provided in First-Strand Synthesis Kit (Takara) and miRNA specific forward primers listed in Suppl Table 3. As with mRNA, miRNA levels were quantified using the 2^ΔΔCt^ method and normalized to miR-103a-3p (set at 100)^71^.

### Western blotting

Cell lysis and western blot analysis was assessed as previously described^33,35,37,57,63^. Proteins were visualized using the ECL Prime detection kit (GE Healthcare). Primary antibodies used include Ezh2 (1:10,000; 5246, Cell Signaling), Actin (1:10,000; sc-1616; Santa Cruz), histone H3 (1:10,000; 05–928; Millipore), and H3K27me3 (1:5000; 17–622; Millipore). Uncropped and additional examples of western blots are shown in Suppl Fig. 3. The blots were cut prior to antibody hybridization. Maximal sizes of the final blots for each antibody are shown in the supplementary data. Where necessary, images were cropped and rotated to ensure alignment of bands in the final figures.

### Alizarin red staining and quantification

Mineral deposition of differentiating MC3T3 cells was assessed by alizarin red staining as previously described^35,37,57,63,72^. Briefly, cell cultures were fixed in 10% neutral buffered formalin and stained with 2% Alizarin Red (Sigma) to visualize calcium deposits. Alizarin red absorption was quantified by ImageJ software^73^.

### Microscopy

Cell viability and visual evidence for miRNA transfection (Cy3 dye-labeled Pre-miRNA Negative Control) was examined using an inverted fluorescent microscope (ZeissAxioVert.A1). Cell viability was assessed using a two-color fluorescence assay based on the simultaneous determination of live (calcein acetoxymethyl [AM]: green) and dead (ethidium homodimer-1: red) cells as suggested by the manufacturer (Molecular Probes)^74,75^ and images obtained with 10 × magnification objective with the Texas red filter. Cells transfected with a Cy3-labeled miRNA negative control were imaged under bright-field and Texas red filter using the 20 × magnification objective to confirm the presence of the fluorescent dye within the transfected cells. All images were processed with the Zen software (2.3 SP1, Zeiss 2015).

### Animal welfare

Mouse studies were performed according to recommendations provided by the National Institutes of Health and the Institute of Laboratory Animal Resources, National Research Council. The Mayo Clinic Institutional Animal Care and Use Committee approved all animal studies. Animals were housed in an accredited facility under a 12-h light/dark cycle and provided water and food ad libitum. Mice were fed either a normal (PicoLab Rodent Diet 20, LabDiet) or a doxycycline-enriched diet (Mod LabDiet® 5053, LabDiet). All studies were done in accordance with Animals in Research: Reporting In Vivo Experiments (ARRIVE) guidelines^76^.

### miR-101a transgenic mice

An unpublished derivative of the mouse embryonic stem (ES) cell line KH2 which supports transgenic expression of mature miR-101a (Stock Number: M000006) was obtained from the National Cancer Institute Mouse Repository (Frederick National Laboratory for Cancer Research, Frederick, MD). The KH2 cell line contains a second-generation reverse tetracycline transactivator (rtTA-M2) that is expressed from the Rosa26 promoter^77,78^. The parental KH2 cell line also contains a specific recombination site (‘ColA’) that permits rapid insertion of transgene expression cassettes using the Flp recombinase^77,78^. The recombination site is located 0.5 kbp downstream of the 3′UTR sequences of the mouse Col1a1 gene in a chromosomal region that is permissive for transcriptional activation in different mammalian species^77,79^. The KH2 cell line was subsequently modified for the systematic analysis of gene function by RNA interference in which any small RNA (shRNA or miRNA) that is incorporated within the body of the precursor for miR-30 can be induced by tetracycline (doxycycline)^47^. The KH2-M000006 cell line used in this study contains a multi-model gene cassette (TRE-GFP-miR-30/101a) in which a tetracycline response element (TRE) supports expression of a mRNA encoding green fluorescent protein (GFP) mRNA and a 3′UTR that contains the precursor scaffold for miR-30 in which the sequences for the mature miR-30 were replaced by the sequences of the mature miR-101a. A diagram clarifying the molecular components of the model is provided as Suppl Fig. 1. The ES cells were injected into C57BL/6 mouse blastocysts to generate chimeric mice. These mice were then bred to obtain germline transmission. The following genotyping primers were used: Col1a1 locus: Forward—AATCATCCCAGGTGCACAGCATTGCGG, Reverse #1—CTTTGAGGGCTCATGAACCTCCCAGG, Reverse #2—ATCAAGGAAACCCTGGACTACTGCG; Rosa locus: Forward #1—AAAGTCGCTCTGAGTTGTTAT, Forward #2—GGAGCGGGAGAAATGGATATG, Reverse—GCGAAGAGTTTGTCCTCAACC. For all experiments, homozygous miR-101a and homozygous Rosa26-rtTA were bred with each other or C57BL/6 wild type mice to generate the three experimental mice used in these studies: Rosa26-rtTA heterozygous mice (R26, *Rosa26*^*rtTA/*+^), mir-101a heterozygous mice (101a, *Col1a1*^*mir-101a/*+^), and Rosa26-rtTA heterozygous + mir-101 heterozygous (R26/101a, *Rosa26*^*rtTA/*+^*: Col1a1*^*mir-101a/*+^).

For post-natal induction of mir-101a (R26/101a cohorts only), breeding pairs and experimental mice were maintained on normal diet. At three weeks of age (weaning), experimental mice were maintained on regular diet or placed on doxycycline-enriched diet. For some mice, the doxycycline diet was replaced with regular diet at six weeks.

For pre-natal induction, all breeding mice were placed on doxycycline-rich diet at least five days before combining male and female breeders. Thus, for experimental mice, which includes all three genotypes (R26, 101a, and R26/101a), doxycycline-rich diet was provided from inception to sacrifice via mother (e.g., blood stream and milk) or by consuming doxycycline rich diet^47–49^.

### μCT analysis

Bones were scanned using a 65 kV source, 0.5 mm Al filter, 0.7° rotation and two-image averaging with an isotropic voxel size of 9 μm using a SkyScan 1176 system (SkyScan)^80^. The terminology and units used for µCT are those recommended by the American Society for Bone and Mineral Research (ASBMR)^81^.

### Bone histomorphometry

Femur from 8-week-old mice were fixed in 10% neutral buffered formalin and embedded using previously established methods at the ICMH Histology and Histomorphometry Core^80^. Sagittal sections of methyl methacrylate-embedded femora were obtained. Osteoclasts were quantified in TRAPase/Toluidine blue-stained and osteoblasts/osteocytes in von Kossa/McNeal-stained sections. Histomorphometric analysis was performed using OsteoMeasure high resolution digital video system (OsteoMetrics Inc.). The terminology and units used are those recommended by the ASBMR Histomorphometry Nomenclature Committee^82^.

### High throughput RNA sequencing and bioinformatic analysis

Total RNA from differentiating MC3T3 cells (days 0, 6, 10, 17, and 24) and mouse calvaria was isolated as described above. To normalize biological variation, three biological samples were pooled (equal amounts of RNA) for each MC3T3 time point. MicroRNAs were sequenced as previously described in detail^71,83,84^. The resulting data were deposited in the Gene Expression Omnibus (GEO) of the National Center for Biotechnology Information (GSE155390). Of note, #1, #2, #3 calvaria within this manuscript correspond to samples p64, p73, and p88, respectively, within the GEO dataset. miRNA expression data are expressed in counts per million (CPM). Hierarchical clustering was performed using Morpheus matrix visualization and analysis software after a Log2 adjustment was made for each miRNA (Broad Institute, Cambridge, MA). Venn Diagrams were generated using Venny 2.1 online tool (BioinfoGP).

### Circulating bone markers

Terminal serum samples were collected at sacrifice and stored at -80C. Serum N-terminal propeptide of type I procollagen (P1NP) (catalog #AC-33F1, Immunodiagnostic Systems Inc.) and C-telopeptide fragments (CTX) (catalog #AC-06F1, Immunodiagnostic Systems Inc.,) were measured as suggested by the manufacturer and previously described^85,86^.

### Statistics

Data are shown as mean ± standard deviation and statistical analysis was performed with unpaired Students t-test or one-way analysis of variance (ANOVA) using Tukey’s multiple comparisons test in GraphPad Prism 9. When the overall ANOVA F-test was significant, subsequent pairwise comparisons were performed using ordinary one-way ANOVA for multiple comparisons. Significance is noted (*p < 0.05; **p < 0.01; ***p < 0.001, and ****p < 0.001).

## Supplementary Information

Supplementary Figure 1.

Supplementary Figure 2.

Supplementary Figure 3.

Supplementary Table 1.

Supplementary Table 2.

Supplementary Table 3.

## Data Availability

The data that support the findings of this study are available in Gene Expression Omnibus (GEO) database at https://www.ncbi.nlm.nih.gov/geo/, Accessions #GSE155390.
