# Supplementary figures and images for: MicroRNA-101a enhances trabecular bone accrual in male mice
Source: Sci Rep. 2022 Aug 3;12:13361. doi: 10.1038/s41598-022-17579-0 (PMC9349183; doi:10.1038/s41598-022-17579-0)

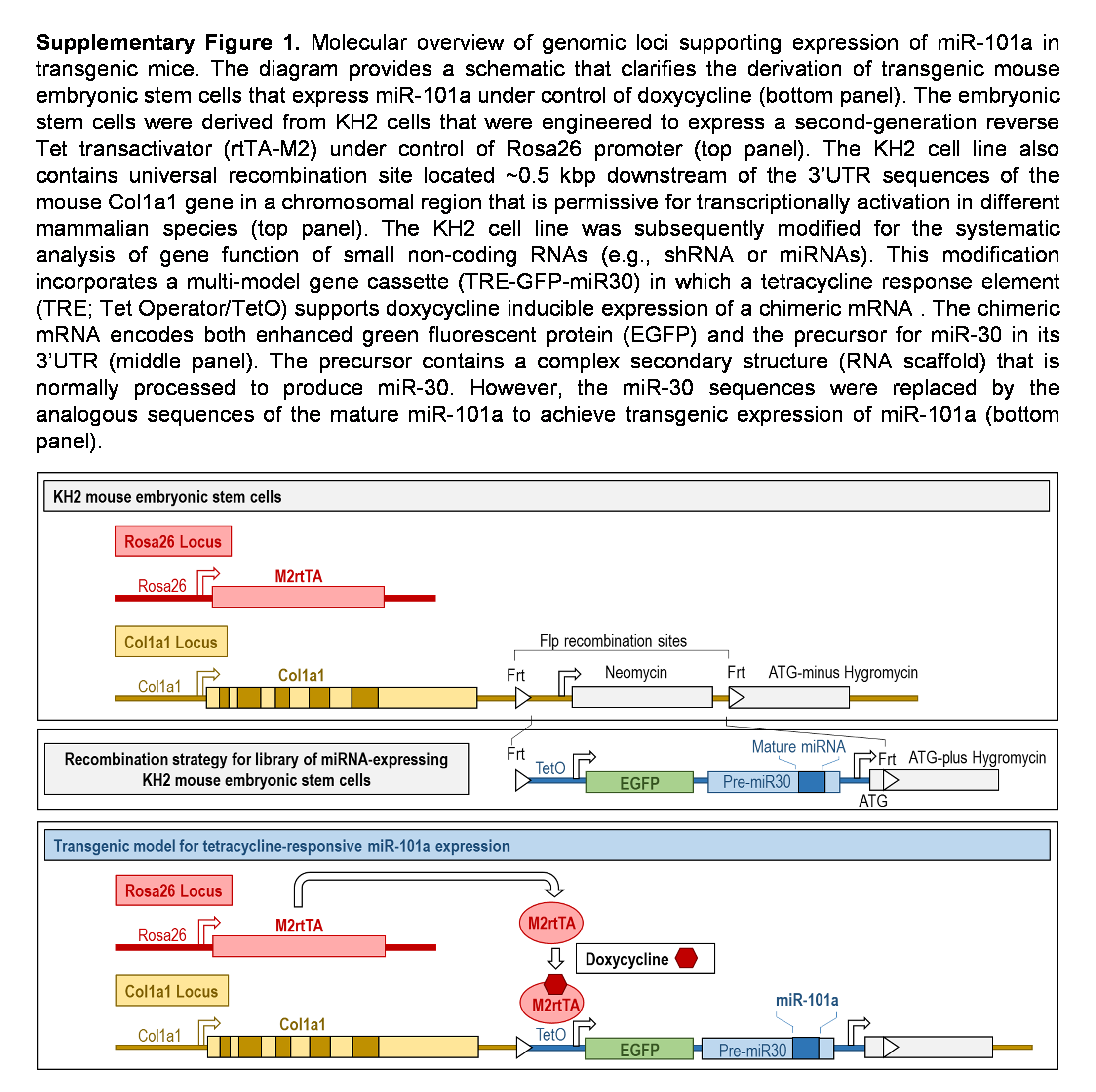

Supplement: Supplementary file 1 — Supplementary Figure 1. [file 41598_2022_17579_MOESM1_ESM.tif]
